# Revisiting Centiloids using AI

**DOI:** 10.21203/rs.3.rs-7015694/v1

**Published:** 2025-07-08

**Authors:** Pierrick Bourgeat, Jurgen Fripp, Leo Lebrat, Ying Xia, Azadeh Feizpour, Timothy Cox, Georgios Zisis, Ashley Gillman, Manu Goyal, Duygu Tosun, Tammie Benzinger, Pamela LaMontagne, Michael Breakspear, Michelle Lupton, Cathy Short, Robert Adam, Joanne Robertson, Reisa Sperling, Sid O’Bryant, Sterling Johnson, Clifford Jack, Christopher Schwarz, Denise (C) Park, Frederik Barkhof, Gill Farrar, Ariane Bollack, Lyduine Collij, Susan Landau, Robert Koeppe, John Morris, Michael Weiner, Victor Villemagne, Colin Masters, Christopher Rowe, Vincent Doré

**Affiliations:** CSIRO; CSIRO; Queensland University of Technology; The Australian e-Health Research Centre, CSIRO Health and Biosecurity; The Florey Institute of Neuroscience and Mental Health; CSIRO; The Florey Institute of Neuroscience and Mental Health; CSIRO; Washington University School of Medicine; University of California, San Francisco; Washington University in St. Louis; Mallinckrodt Institute of Radiology, Washington University School of Medicine; University of Newcastle; QIMR Berghofer Medical Research Institute; Central Adelaide Local Health Network Memory Service and Memory Clinical Trials; University of Queensland; The Florey Institute of Neuroscience and Mental Health; Harvard Aging Brain Study, Department of Neurology, Massachusetts General Hospital, Boston, MA 02114;Center for Alzheimer Research and Treatment, Department of Neurology, Brigham and Women’s; University of North Texas Health Science Center; wisc; Mayo Clinic; Mayo Clinic; University of Texas, Dallas; VU University Medical Center; GE HealthCare; GE HealthCare; Clinical Memory Research Unit, Department of Clinical Sciences Malmö, Lund University, Lund, Sweden; University of California Berkeley; University of Michigan; Knight Alzheimer Disease Research Center; University of California-San Francisco; University of Pittsburgh; florey; Austin Health; Austin Health

## Abstract

The Centiloid scale is the standard for Amyloid (Aβ) PET quantification, widely used in research, clinical settings, and trial stratification. However, variability between tracers and scanners remains a challenge. This study introduces DeepSUVR, a deep learning method to correct Centiloid quantification, by penalising implausible longitudinal trajectories during training. The model was trained using data from 2,098 participants (6,762 Aβ PET scans) in AIBL/ADNI and validated using 15,806 Aβ PET scans from 10,543 participants across 10 external datasets. DeepSUVR increased correlation between tracers, and reduced variability in the Aβ-negatives. It showed the strongest association with cognition, highest AUC against visual reads and best longitudinal consistency between studies. DeepSUVR also increased the effect size for detecting lower Centiloid increase per year in the A4 study. DeepSUVR advances Aβ PET quantification, outperforming standard approaches, which is particularly important for consistent decision making and to detect subtle and early changes in clinical interventions.

## Introduction

The Centiloid scale was developed to unify the quantification derived from all β-amyloid (Aβ) PET tracers onto a single, standardised scale (Klunk et al., 2015). It has since been widely adopted as the default quantification scale in Ab PET research and serves as a secondary end-point in anti-Aβ clinical trials (Dyck et al., 2023; Sperling et al., 2023). Accurate Centiloid quantification is crucial for several applications: (1) reducing diagnosis uncertainty, especially in cases with equivocal visual PET assessments; (2) informing patient inclusion criteria for anti-Ab disease-modifying therapies, particularly when a specific Aβ burden threshold is required to target distinct disease stages; (3) accurately quantifying Aβ clearance during therapeutic interventions; and (4) supporting the identification of early or emerging Aβ pathology (Collij et al., 2024a). While the Centiloid framework has significantly advanced the standardisation of Aβ quantification across diverse studies and tracers (Shekari et al., 2024), its current standard implementation is predicated on the assumption that its proposed reference and target masks used are universally optimal. This approach may overlook variability introduced by different PET tracers, scanners or image reconstruction parameters; variability that becomes apparent in head-to-head studies and longitudinal studies (Bourgeat et al., 2022; Gillman et al., 2025).

Prior to the Centiloid, different groups established tracer-specific optimal reference and target regions for calculating the Standardised Uptake Value Ratio (SUVR). While the target regions exhibited considerable similarity across tracers, the selection of an optimal reference region varied significantly. The cerebellum cortex (Cb) was selected early on for ^11^C-PiB (PIB) due to its sparse fibrillar plaques pathology (Klunk et al., 2004). It was later adopted for ^18^F-Florbetaben (FBB) (Becker et al., 2013) and ^18^F-NAV4694 (NAV) (Rowe et al., 2013). For ^18^F-Flutemetamol (FMM), the pons was identified as giving the best agreement with histopathology after evaluation of several candidate regions (Thurfjell et al., 2014). For ^18^F-Florbetapir (FBP), the whole cerebellum (WCb) was chosen because its mixture of gray matter (GM) and white matter (WM) was thought to reflect the mixed tissue composition often present in the neocortical target mask (Fleisher et al., 2011). However, the WCb later proved suboptimal for detecting longitudinal changes with FBP, and a composite reference region, composed of an eroded subcortical WM mask+WCb, was found to be superior for both longitudinal (Landau et al., 2015) and cross-sectional analyses (Bourgeat et al., 2022), a finding that was also replicated in PIB (Schwarz et al., 2017). Lastly, while the Centiloid neocortical mask was defined using a data-driven approach, by subtracting an average Ab-negative image from an average Ab positive image to isolate regions of tracer retention, the reference mask was based on anatomical delineation, a method inherently more susceptible to subjectivity and potential suboptimality.

Data-driven approaches have been proposed to improve the Centiloid quantification, including the Aβ load (Whittington and Gunn, 2019), Aβ index (Leuzy et al., 2020) and the non-negative matrix factorisation (NMF) (Bourgeat et al., 2021). They all rely on the decomposition of the image into a specific and non-specific component, thereby reducing the influence of the reference region (with Aβ index being the only approach to be truly reference region-free). A recent comparison showed that they provide a strong association with the binding potential, which is often considered a more direct estimate of the underlying Aβ burden and can potentially reduce the number of participants required in simulated prevention trial scenarios compared to the standard Centiloid method (Bollack et al., 2023a). Despite these advantages, broader adoption of these methods is hindered by limited general availability, limited validation, and reliance on fixed preprocessing pipelines.

A few approaches have been proposed to leverage the power of deep learning (DL) for Centiloid quantification. These strategies typically either replace the conventional spatial normalisation to a template, as used in the standard pipeline, with a DL-based approach (Kang et al., 2023) or directly estimate the Centiloid (Yamao et al., 2024) or amyloid positivity status (Fan et al., 2024) from the raw PET images. However, because these DL methods often emulate the standard pipeline’s methodology or are trained to reproduce its quantification, they tend to inherit the same limitations as the original Centiloid framework.

This study hypothesises that sub-optimal reference and/or target regions are the main sources of variability in longitudinal Centiloid measurements, and given that SUVR is a ratio of the tracer retention in these two regions, a SUVR correction factor could therefore mitigate variability originating from either region. We further hypothesise that such a correction factor could be estimated by penalising deviations from expected longitudinal trajectory of amyloid accumulation. The natural history curve for Ab accumulation, initially conceptualised by Jack and colleagues (Jack et al., 2010), has been independently characterised by multiple research groups (Betthauser et al., 2022; Bollack et al., 2024; Jack et al., 2013; Jagust et al., 2021; Schindler et al., 2021; Villemagne et al., 2013). This model implies that an individual’s current Centiloid value is predictive of their future Centiloid changes. Within a machine learning framework, this natural history curve of Ab accumulation can serve as a temporal prior, allowing deviations from this model to be penalised during model training. We further hypothesise that the resulting DL-corrected SUVR can be used to define new, data-driven reference and target masks that can be used for both explainability of the DL network and as simplified implementation for use in existing pipelines. This can be formulated as an optimisation task, wherein new reference and target masks are optimised across all tracers to ensure the resulting SUVR exhibits the highest possible correlation with the DL-corrected SUVR.

In this work, we developed and trained a DL model, called DeepSUVR, using observational longitudinal data to predict a SUVR correction factor, which once transformed into Centiloid, minimises deviations from the established amyloid natural history curve. DeepSUVR was trained using 5-fold cross-validation on the combined AIBL and ADNI dataset. It was subsequently validated on the cross-sectional data from the GAAIN Centiloid calibration, ADNeT, ADNI DOD, PISA datasets, as well as on the longitudinal data from A4-LEARN, AMYPAD, DLBS, HABS-HD, MCSA, OASIS3, WRAP cohorts, and the held-out folds from the AIBL+ADNI datasets. The corrected SUVRs were then used to generate novel reference and target mask based on the combined AIBL+ADNI dataset. The resulting Centiloids are compared against the Standard Centiloid $\left(\text{C}{\text{L}}_{\text{Std}}\right)$, Centiloids computed using the composite reference region for FBP $\left(\text{C}{\text{L}}_{\text{Comp}}\right)$, and our previously proposed NMF approach (Bourgeat et al., 2021) ${(\text{CL}}_{\text{NMF}})$, across both the training and testing cohorts.

The resulting DeepSUVR model and derived masks are freely available for non-commercial use and can be downloaded from https://github.com/csiro/DeepSUVR.

## Results

### Datasets

The demographics characteristics of the training (AIBL, ADNI) and independent testing cohorts are detailed in Supplementary Table 1. The dataset for model development was derived from the combined AIBL and ADNI cohorts, including a total of 9,266 amyloid PET scans from 3,994 unique participants (3,909 FBP, 1,747 PIB, 902 FBB, 539 FMM and 2,169 NAV). For the model training set, we selected a subset of 6,762 longitudinal PET scans from 2,098 of these AIBL/ADNI participants with two or more PET visits, with scans acquired prior to November 2022 (3,083 FBP, 1,597 PIB, 395 FBB, 487 FMM and 1,200 NAV). Data from the remaining 1,896 AIBL/ADNI participants, who only had a single PET imaging timepoint, were allocated to the internal test set, which was kept separate and not combined with the independent testing cohorts. The independent testing dataset included 15,807 PET scans (3,957 FBP, 5,201 PIB, 4,926 FBB, 1,275 FMM and 448 NAV) from 10,543 unique participants from 10 external cohorts.

### Cross-sectional analysis

The Centiloids obtained using DeepSUVR ${(\text{CL}}_{\text{DS}})$ were strongly correlated with the Centiloids obtained from the standard method $\left(\text{C}{\text{L}}_{\text{Std}}\right)$ for all tracers in both the training cohort ${(\text{R}}^{2}>0.91)$ and the testing cohorts $\left({\text{R}}^{2}>0.85\right)$ (Supplementary Figure 1). The correlation was strongest for NAV, followed by PIB, FBB, FMM and was weakest for FBP.

### Head-to-head tracer comparison

**Figure 1:**
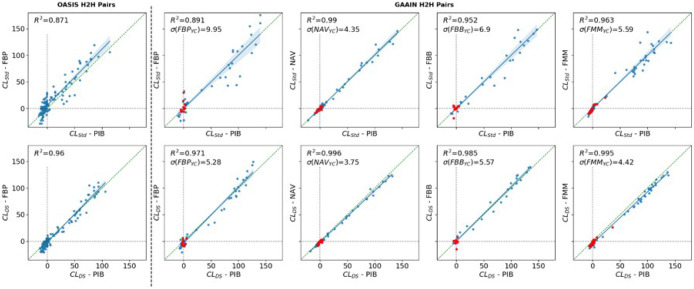
Scatterplots comparing PiB vs FBP in the OASIS Head-to-Head (H2H) dataset (left) and each ^18^F-Tracer versus PIB in the GAAIN Head-to-Head Calibration dataset (Right), using ${\text{CL}}_{\text{Std}}$ (top) and ${\text{CL}}_{\text{DS}}$ (bottom). The correlation between each pair of tracers is assessed using the coefficient of determination ${\text{R}}^{2}$. The standard deviation in the Young Controls (YC) for each tracer is denoted as $\left.\sigma ({\text{Tracer}}_{\text{YC}}\right)$. Note that for FMM, one YC with a CL>20 on both PIB and FMM across all methods was determined to be an outlier and excluded from the standard deviation calculation.

Using data from 120 OASIS study participants with repeat PIB and FBP scans acquired within 7 months of each other, and from 289 GAAIN Calibration dataset participants scanned with PiB and one of the ^18^F tracers, the scatterplot in Figure 1 illustrates the agreement between each ^18^F tracer and PIB. $CL_{DS}$ demonstrated the highest correlation between each ^18^F tracer and PIB; within the GAAIN Calibration dataset, all tracers achieved an ${\text{R}}^{2}$ above 0.97 with PIB. Compared to the standard method, the greatest improvement in the coefficient of determination was observed with FBP (from ${\text{R}}^{2}=0.89$ to ${\text{R}}^{2}=0.97$), followed by FBB (${\text{R}}^{2}=0.95$ to ${\text{R}}^{2}=0.98$) and FMM (${\text{R}}^{2}=0.96$ to ${\text{R}}^{2}=0.99$) and with a marginal improvement for NAV (${\text{R}}^{2}=0.990$ to ${\text{R}}^{2}=0.996$). A similar improvement in ${\text{R}}^{2}$ between FBP and PIB was observed in the OASIS head-to-head dataset, increasing from 0.87 to 0.96. None of the other evaluated methods achieved higher agreement between any pair of tracers (supplementary Fig 2). Furthermore, the standard deviation in the young controls was smallest using DeepSUVR for all tracers, except for FBB, where the NMF method achieved the lowest standard deviation.

### Agreement with Visual Reads

The area under the curve (AUC) values, reflecting agreement between visual PET reads (from ADNI, ADNI-DOD, A4, HABS-HD and AMYPAD) and the positivity based on Centiloid values obtained using each quantification method, are presented in Table 1. Although the AUC values were high for all quantification methods, $CL_{DS}$ consistently yielded the highest AUC across all cohorts, with significant improvement over $CL_{Std}$ in A4, ADNIDOD, HABS-HD and AMYPAD FMM.

### Agreement with Neuropathology

The effect size (ES; Cohen’s d) between FBP CLs from ADNI participants, stratified by CERAD (Mirra et al., 1991) diffuse plaques scores (‘none/sparse’ vs. ‘moderate/frequent’), are presented for each quantification method in Supplementary Figure 3. While the ES were high for all quantification methods, the highest ES was obtained using $CL_{DS}$, and was significantly higher than the one obtained using $\text{C}L_{Std}$ (Cohen’s d=3.70 vs 2.79, p<0.001).

### Baseline Centiloid distribution

**Figure 2:**
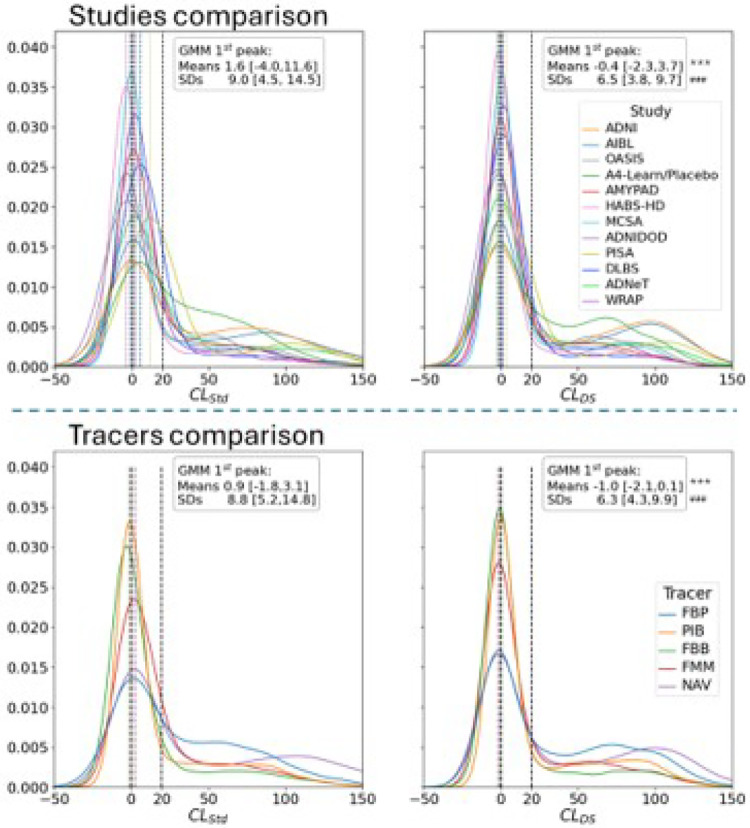
Histogram distribution of Centiloid values across the 12 cohorts using the Standard and DeepSUVR CL methods. For each approach, a Gaussian mixture is fitted to the distribution of Centiloid values of each study (top) and each tracer (bottom), and the average mean [min,max] and average standard deviation [min,max] of the first peak across all studies (tracer respectively) is reported. The dashed lines mark the 0CL and 20CL. Significantly lower variability in the means and standard deviations when comparing ${\text{CL}}_{\text{DS}}$ to ${\text{CL}}_{\text{Std}}$ across studies/tracers based on bootstrapping are indicated using: *** (p<0.001) for lower variabilities in the means and ### (p<0.001) for lower variabilities in the standard deviations.

The distributions of the baseline Centiloid values computed using the Standard method and DeepSUVR across all studies and tracers are presented in Figure 2. To assess the variability of the Aβ-negative across studies, a two-Gaussian mixture model was fitted to each distribution. The average mean and standard deviation of the first peak (used as a proxy of the Aβ-negative Centiloid distribution), averaged across all studies, are reported in Figure 2. ${\text{CL}}_{\text{DS}}$ showed significantly more consistent means for the first peak, with all peaks contained within a 6 CL unit range [−2.3CL to 3.7CL] across studies, compared to a 15 CL unit range when using ${\text{CL}}_{\text{Std}}[-4.0\text{CL}\;\text{to}\;11.6\;\text{CL}]$. Similarly, ${\text{CL}}_{\text{DS}}$ showed a significantly smaller standard deviation in the Aβ-negative peak with an average standard deviation of 6.5 (range of [3.8, 9.7]) compared to 9.0 (range of [4.5, 14.5]) for ${\text{CL}}_{\text{Std}}$. The distributions computed using the other quantification approaches are presented in Supplementary Figures 4 and 5, showing that using the ${\text{CL}}_{\text{Comp}}$ and ${\text{CL}}_{\text{NMF}}$ both led to some reduction in variability in the Aβ-negative compared to ${\text{CL}}_{\text{Std}}$, it was still much higher than with ${\text{CL}}_{\text{DS}}$.

### Correlation with Cognitive measures

Spearman rank correlations between baseline Centiloid values and mini-mental state examination (MMSE) scores in the external testing cohorts are reported in Supplementary Table 2. The strongest association between Centiloid and MMSE was obtained using ${\text{CL}}_{\text{DS}}$ (r=−0.111) which was significantly stronger than ${\text{CL}}_{\text{Std}}$ (r=−0.077, p<0.001). Similarly, the largest effect sizes (Cohen’s d) between CDR 0 vs 0.5, CDR 0.5 vs 1 and CDR 0 vs 1 were all achieved using ${\text{CL}}_{\text{DS}}$ (0.303, 0.718 and 1.061, respectively), which were all significantly higher than ${\text{CL}}_{\text{Std}}$ (0.272, 0.644 and 0.960, respectively, all with p<0.001). Similar findings were obtained in the training cohorts (Supplementary Table 3).

### Longitudinal trajectories

**Figure 3:**
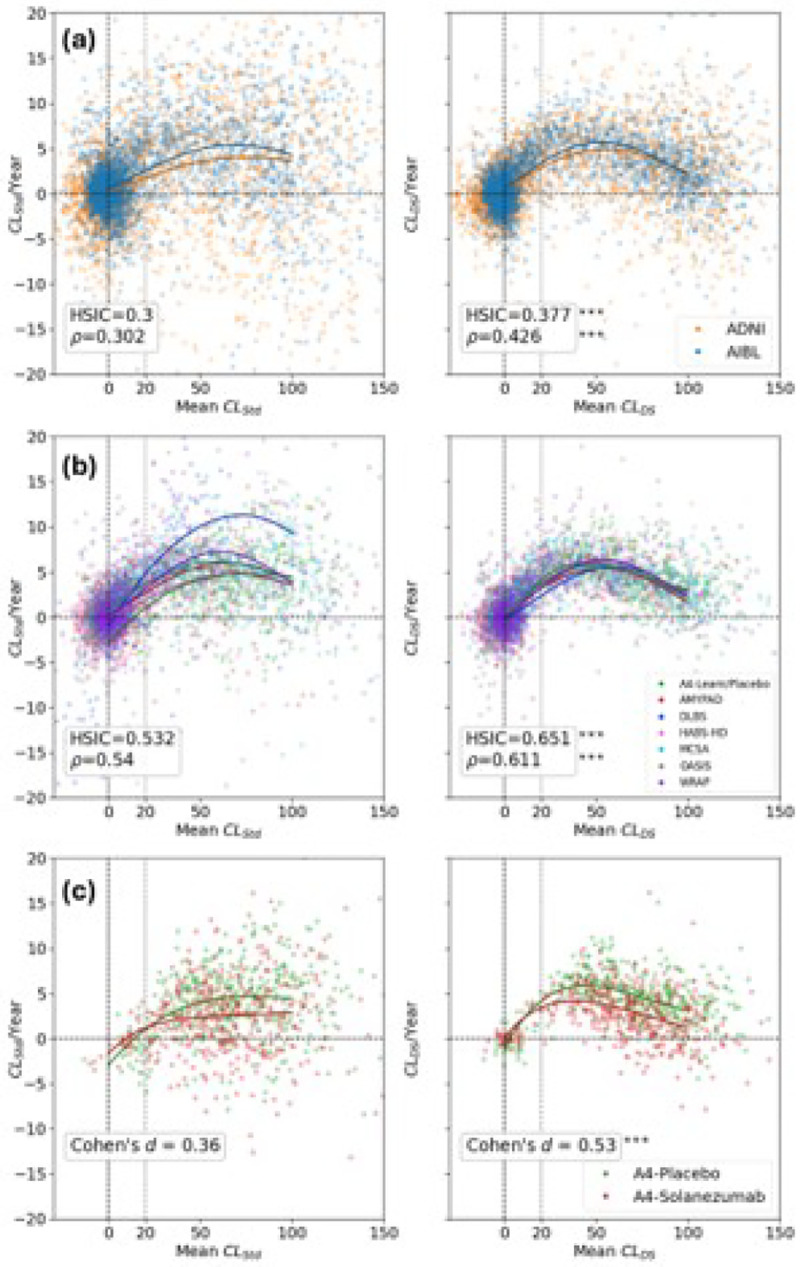
Rate of CL change per year compared to mean CL computed from the 2 training (AIBL/ADNI) cohorts (a.), the 7 testing cohorts with longitudinal data (A4-Placebo, AMYPAD, HABS-HD, OASIS, DLBS, MCSA, WRAP) (b.) and the placebo and treatment arms of the A4 Study (c.) using the Standard (left) and DeepSUVR CL (right) methods. Each point represents the mean and rate of change between a pair of consecutive visits from the same participant. Each curve shows a 5th order polynomial fitted to each study. The Spearman rank correlation between Mean CL and CL/Year is denoted using r (a. and b.), while the effect size of the CL accumulation per year between the 2 arms of the A4 study is denoted using Cohen’s d (c.). The vertical dashed lines mark the 0CL and 20CL, while the horizontal dashed line mark the 0CL/Year. Significantly higher correlation or effect size when comparing ${\text{CL}}_{\text{DS}}$ to ${\text{CL}}_{\text{Std}}$ across studies/tracers based on bootstrapping are indicated using: *** p<0.001.

Rate of annual CL change (CL/Year) versus mean CL values for pairs of consecutive visits, using the Standard and DeepSUVR CL quantifications, for the training and testing cohorts are presented in Figure 3.a and Figure 3.b, respectively. A comparison across all quantification methods is provided in Supplementary Figures 6 and 7. The Hilbert-Schmidt Independence Criterion (HSIC), which is used assess non-linear statistical dependence between the mean CL and CL/Year was the highest for DeepSUVR in both training (HSIC=0.377) and testing cohorts (HSIC=0.651) and were significantly higher than those from ${\text{CL}}_{\text{Std}}$ (HSIC=0.300 in training, HSIC=0.532 in testing, p<0.001), with similar findings using Spearman rank correlation r. To visually assess the concordance between the participants’ longitudinal trajectories in each study, a 5^th^ order polynomial was fitted to each study, and the resulting trajectory plotted in Figure 3. Those curves further emphasise that the trajectories derived from each cohort are more concordant when using DeepSUVR. Additionally, the distribution of annual $CL_{\text{Std}}$ change from consecutive AIBL PiB scans acquired on the same scanner was used to establish a 95% confidence interval for expected annual changes when no change of scanner or tracer occurs. The resulting threshold of −5.8CL/Y and 11.2CL/Y were used to assess the percentage of longitudinal outliers in both the training and testing datasets and are presented in Table 2, showing a 3- to 5-fold reduction in the percentage of outliers when using DeepSUVR compared to the Standard CL in both the training and testing cohorts. Spaghetti plots, with negative outlier trajectories marked in red and positive outlier trajectories marked in blue, are presented in Supplementary Fig 10 for the training set and Supplementary Fig 11 for the testing set.

### A4 Study

Longitudinal trajectories for the treatment and placebo arms of the A4 study, between the 2-month and 56-month visits, using the Standard and DeepSUVR CL quantifications, are presented in Figure 3.c, along with the effect size for the difference in annual CL accumulation between the two arms. A comparison across all quantification methods is shown in Supplementary Figure 8, and a violin-plot of the rate of change in Supplementary Figure 9. The largest effect size was obtained using DeepSUVR (Cohen’s d=0.53), which was significantly higher than the one obtained using $\text{C}{\text{L}}_{\text{Std}}$ (Cohen’s d=0.36, p<0.001). Notably, participants with an Aβ-negative baseline scan, defined as CL<20 using each method’s CL value, exhibited significantly less variability (lower standard deviation) in their rate of change using DeepSUVR $\left(\text{SD}\left(CL_{DS}/\right.\right.\text{Year})=4.96$ compared to $\text{SD}\left(CL_{Std}/\right.\text{Year}\left.)=7.77,p<0.05\right)$.

### Detecting emerging Aβ pathology

This analysis aimed to quantify the proportion of participants with a baseline Aβ-negative scan who subsequently progressed to Aβ-positive status. Using a conservative CL threshold of 30 to define positivity (Collij et al., 2024b; Farrar et al., 2025), participants with at least 4.5 years of follow-up and a baseline Aβ-negative scan (CL<30), were classified as “Emergent Aβ+” if at least one follow-up scan was Aβ-positive (CL≥30); otherwise, they were classified as “stable Aβ-negative”. Using five CL brackets between 5 and 30CLs, the proportion of emergent Aβ-positive participants with a baseline CL within each bracket is presented in Supplementary Table 4 for the testing set and Supplementary Table 5 for the training set. All participants with a baseline ${\text{CL}}_{\text{DS}}$ between 20 and 30 were classified as emergent Aβ-positive in training set and 98% in the testing set. In contrast, using ${\text{CL}}_{\text{Std}}$, only 85% of the participants in the Training set and 69% in the Testing set were emergent Aβ-positive, with the remaining classified as stable Aβ-negative. In the 15–20CL bracket, 81% and 91% were emergent Aβ-positive using $\text{C}{\text{L}}_{\text{DS}}$ in training and testing cohorts respectively, compared to 48% and 69%, respectively, using ${\text{CL}}_{\text{Std}}$. The trajectories of the emerging Aβ-positive and stable Aβ-negative for all methods are presented in Supplementary Fig 12 for the testing cohorts and Supplementary Fig 13 for the training cohorts, further illustrating the reduced variability in the ${\text{CL}}_{\text{DS}}$ longitudinal trajectories.

### DeepSUVR derived masks

**Figure 4:**
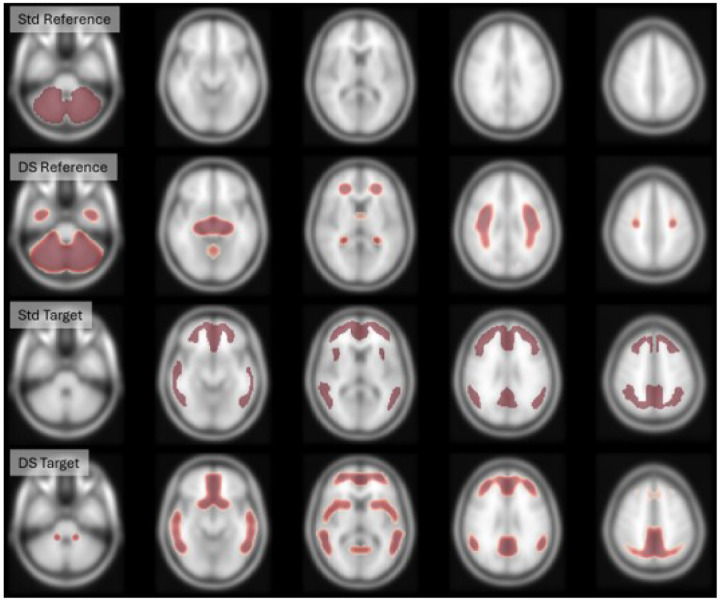
Axial views of the Standard CL reference mask (row1) and the new reference mask derived from DeepSUVR (row2), the Standard CL target mask (row3) and the new target mask derived from DeepSUVR (row4)

Novel reference and target masks were optimised by maximising their correlation with the DeepSUVR-derived SUVRs from AIBL+ADNI dataset across all five tracers (axial views shown in Figure 4, with coronal and sagittal views shown in supplementary Figures 14 and 15, respectively). The DeepSUVR-derived target mask showed a high degree of overlap with the standard Centiloid neocortical mask (Dice coefficient=0.67). In contrast, the derived reference mask, while including the whole cerebellum, also included extensive regions of the subcortical white matter, resulting in less overlap with the standard reference mask (Dice coefficient= 0.50). The Centiloids values derived using these new masks $\left(\text{C}{\text{L}}_{\text{DS}\;\text{mask}}\right)$ showed a very high correlation with $\text{C}{\text{L}}_{\text{DS}}$ across all tracers, ranging from ${\text{R}}^{2}=0.987$ to 0.995 in the training dataset and ranging from ${\text{R}}^{2}=0.983$ to 0.997 in the testing datasets (Supplementary Fig 16). The Centiloid transforms used to convert the ${\text{SUVR}}_{\text{DS}\;\text{mask}}$ into ${\text{CL}}_{\text{DS}\;\text{mask}}$ are listed in Supplementary Table 6.

Although these new masks did not exactly replicate the performance of direct DeepSUVR quantification, they generally performed similarly to DeepSUVR and significantly outperformed ${\text{CL}}_{\text{Std}}$ in all evaluations where ${\text{CL}}_{\text{DS}}$ significantly outperformed ${\text{CL}}_{\text{Std}}$. Specifically, in the GAAIN Calibration dataset, $CL_{DS\;\text{mask}}$ showed very high correlation between each ^18^F tracer and PIB, with all tracers showing a ${\text{R}}^{2}$ above 0.95, and a low standard deviation in the young controls (< 6.2) (Supplementary Figure 2). It also yielded the second highest AUC for agreement with visual reads across all studies (Supplementary Table 7) and the second highest effect size for discriminating CERAD none-sparse and moderate-frequent stages (Supplementary Figure 3). For baseline Centiloid distribution, $CL_{DS\;\text{mask}}$ had the second lowest average standard deviation across studies ($CL_{DS\;\text{mask}}7.7$ vs $CL_{\text{DS}}6.5$) and across tracers ($CL_{DS\;\text{mask}}7.4$ vs $CL_{DS}6.3$) (Supplementary Figure 4 and 5 respectively). It also achieved the second highest correlation with MMSE ($CL_{DS\;\text{mask}}\text{r}=-0.093$ vs $CL_{DS}\text{r}=-0.111$), the second largest effect size between CDR 0 and 0.5 ($CL_{DS\;\text{mask}}0.285$ vs $CL_{DS}0.303$), the largest effect size between CDR 0.5 and 1.0 ($CL_{DS\;\text{mask}}0.727$ vs $CL_{DS}0.718$), and second largest effect size (ES) between CDR 0 and 1.0 ($CL_{DS\;\text{mask}}\text{ES}=1.055$ vs $CL_{DS}\text{ES}=1.061$) (Supplementary Table 2).

When used for longitudinal analysis, $CL_{DS\;\text{mask}}$ had the third highest HSIC in the training ($CL_{DS\;\text{mask}}\text{HSIC}=0.375$ vs $CL_{DS}\text{HSIC}=0.377$) and second highest in the testing cohorts ($CL_{DS\;\text{mask}}\text{HSIC}=0.635$ vs $CL_{DS}\text{HSIC}=0.651$) (Supplementary Fig 6 and 7 respectively). This was also associated with the second lowest percentage of longitudinal outliers (Supplementary Table 8). In the A4 dataset, $CL_{DS\;\text{mask}}$ led to the second highest effect size between the 2 arms of the study ($CL_{DS\;\text{mask}}\text{ES}=0.52$ vs ES=0.53) (Supplementary Figure 8).

## Discussion

In this work, we present DeepSUVR, a deep learning technique designed to learn a SUVR correction factor to improve the longitudinal consistency of Aβ PET measurements, thereby improving inter-tracer agreement. This work also addresses a machine learning challenge of improving quantification in scenarios where definitive ground truth is unavailable, or where the available ground truth may be noisy or biased. The novelty of this approach lies in leveraging expected population-level longitudinal amyloid trajectories to correct the measurements that constitute these trajectories. By limiting the use of the longitudinal information to the loss function during training, the resulting network does not require longitudinal data at inference time, significantly broadening its applicability and reducing the risk of overfitting to specific longitudinal trajectories. Though specifically applied here to Aβ PET, this approach might be of broad value for other aging related biomarkers where ground truth is not readily available but longitudinal trajectories are expected to be constrained.

Another significant contribution of this work is to address the black-box nature of the deep learning algorithm used. By optimising new reference and target masks that largely emulate DeepSUVR’s corrected quantification, and demonstrating their use in quantification in external datasets, provides a tangible means to interpret the model’s impact. While this is an indirect explainability, as it does not attempt to directly understand the model’s decisions such as saliency or activation maps, it provides a practical and easy-to-understand approach to interpretability. Another benefit of the masks is that they could be more easily integrated into existing pipelines, while providing most of the performances of the full DeepSUVR network.

DeepSUVR demonstrated reduced intra- and inter-study variability both cross-sectionally (evidenced by lower variance in the Aβ-negative peaks across studies and tracers) and longitudinally (indicated by increased HSIC and Spearman rank correlation of the longitudinal trajectories with fewer outliers). A beneficial consequence of this reduced variability was an increased inter-tracer agreement in both the GAAIN and OASIS head-to-head datasets, as well as a reduction in the variance in the GAAIN young controls. These improvements collectively contributed to a narrower distribution of the Aβ-negative group across all cohorts. The increasing reliance on Centiloid values for clinical diagnosis and treatment eligibility underscores the critical need for improved concordance among different tracers to ensure consistent decision-making. An Aβ quantification that is more sensitive to longitudinal changes will also improve monitoring of therapeutic responses. With an increasingly large body of work trying to model the natural history of Aβ accumulation in relation to different covariates or other biomarkers, improving the concordance between studies as well as reducing their longitudinal variability also means that we will be able to better combine and harmonise large datasets, increasing the power to detect subtle effects on amyloid accumulation rates or age of onset.

The inherent non-linearity and ‘black-box’ nature of deep-learning technique raise valid concerns on potential model overfitting and removal of biological variability in the participants’ trajectories. Such effects could obscure true differences between sub-groups (such as based on *APOE* status, sex, age, ethnicity, etc…), and prove detrimental to understanding differences in response to therapy (Andrews et al., 2025). Our simulation study presented in supplementary materials showed that if a small proportion of the population within the training data exhibited a distinct rate of accumulation, the DeepSUVR model largely preserved this unique trajectory rather than forcing conformity with the majority pattern. These results indicate that DeepSUVR primarily mitigates variability stemming from noise, errors in spatial normalisation or differences in tracers and scanner, rather than suppressing participants’ inherent biological variability. These findings were further supported by our analysis on the A4 study, where distinct trajectories between the placebo and treatment arms were observed, with DeepSUVR providing the largest difference in their respective rates of change.

Although the relationship between Ab burden and cognition is typically considered indirect, the increased correlation with MMSE, and increased effect size between CDR 0, 0.5 and 1 provide further evidence that DeepSUVR may be providing a more accurate estimate of Ab burden.

### Limitations

While this study used 2 large datasets for training and 10 for testing, one of the main limitations is the imbalanced representation of PET tracers in the datasets. In particular, FBP was over-represented in the training set (45%), while PIB, FBB, and FBP were most common in the testing set (33%, 31% and 25%, respectively). While NAV was well represented in the training set (23%), its representation in the external datasets was limited (3%), and no external study had longitudinal NAV. DeepSUVR’s performance in external NAV datasets therefore needs to be further explored.

The use of different PET scanners has also started to emerge as a potential factor affecting quantification (Bollack et al., 2023b; Gillman et al., 2025). In this work, we attempted to alleviate the impact of using different scanners by using random smoothing as part of the training scheme. While this can account for differences in resolution, it cannot account for differences due to other factors such as scatter correction and different reconstruction methods (Gillman et al., 2025). DeepSUVR could potentially be trained to use scanner information, but this is not trivial since 32 scanners were used in the training set alone, with different reconstruction parameters, and with only a handful present in the testing sets, raising the question of how to generalise the model to unobserved scanners. Scanners and tracers are also often well correlated, as sites tend to favour a single tracer per scanner, making it harder to disambiguate the scanner effect from the tracer effect. One potential approach could be to use a simplified grouping based on the scanner technology, focusing on factors that are most likely to be driving significant differences such as analogue vs digital detector, PET-CT vs PET-MR, or different type of reconstructions, such as Time-of-Flight (ToF) vs non-ToF. This was however outside the scope of this current work.

In conclusion, this work demonstrates that deep learning provides a significant improvement in PET quantification of Ab burden, outperforming standard methods both cross-sectionally and longitudinally in both observational and interventional studies. DeepSUVR enhances the harmonisation of large datasets and different PET tracers, while also reducing longitudinal variability. This will allow better pooling of datasets and improve our ability to detect differences in trajectories between subgroups. A superior Centiloid harmonisation will also allow researchers to better validate and compare existing and novel plasma and other biomarkers for Aβ. With the advent of disease modifying therapies, Aβ PET is gaining an increasingly important role for both qualifying patients for treatment and as an outcome measure. More robust and harmonised quantification will allow consistent decision making across centres. Additionally, the reduction in longitudinal variability will also be critical in trials where the outcome measure of an intervention is expected to be subtle such as altering the rate of accumulation.

## Methods

### Data

Data used in this study combined eleven imaging studies in Alzheimer’s disease: the Australian Imaging, Biomarkers and Lifestyle Study of ageing [AIBL] (Ellis et al., 2009), Alzheimer’s Disease Neuroimaging Initiative [ADNI] (Petersen et al., 2010), the Open Access Series of Imaging Studies [OASIS3] (LaMontagne et al., 2019), the Anti-Amyloid Treatment in Asymptomatic Alzheimer’s [A4] Study and Longitudinal Evaluation of Amyloid Risk and Neurodegeneration [LEARN] Study (Sperling et al., 2020), the Amyloid imaging to prevent Alzheimer’s disease [AMYPAD] (Collij et al., 2023; Frisoni et al., 2019; Lopes Alves et al., 2020), the Mayo Clinic Study of Aging [MCSA] (Roberts et al., 2008), the Health and Aging Brain Study: Health Disparities [HABS-HD] (O’Bryant et al., 2022), the Dallas Lifespan Brain Study [DLBS] (Park et al., 2024), the Wisconsin Registry for Alzheimer’s Prevention [WRAP] (Johnson et al., 2017), the Department of Defense Alzheimer’s Disease Neuroimaging Initiative [ADNI-DOD] (Weiner et al., 2014), the Prospective Imaging Study of Ageing [PISA] (Lupton et al., 2021) and the Alzheimer’s Disease Network [ADNeT] (Lin et al., 2020).

Data used in the preparation of this article were partly obtained from the Alzheimer’s Disease Neuroimaging Initiative (ADNI) database (adni.loni.usc.edu). The ADNI was launched in 2003 as a public-private partnership, led by Principal Investigator Michael W. Weiner, MD. The primary goal of ADNI has been to test whether serial magnetic resonance imaging (MRI), positron emission tomography (PET), other biological markers, and clinical and neuropsychological assessment can be combined to measure the progression of mild cognitive impairment (MCI) and early Alzheimer’s disease (AD). For up-to-date information, see www.adni-info.org.

### Visual reads

In A4, 92 scans were read by two readers local to the imaging site. For 12 scans where the reads were discordant, the consensus between the two readers was used. The remaining 1680 scans were read by a single reader. In ADNI and ADNI-DOD, a single reader performed an initial visual read. For challenging cases and in cases where the visual read and the quantification were discordant, at least three readers performed a consensus review of the scans. When available, the consensus review was used. Otherwise, the initial read was used. In HABS-HD, the PET images were interpreted by licensed fellowship trained radiologists specialising in Nuclear Neuro-based imaging recognising Amyloid and Tau positivity. In AMYPAD, images were rated, together with a T1-weighted MRI scan or CT scan, as either positive (binding in 1 or more cortical brain region unilaterally, or striatum in the case of FMM) or negative (predominantly white matter uptake)(Collij et al., 2025).

### Preprocessing

The preprocessing of the PET images was previously described (Bourgeat et al., 2022), except that in this work, there was no smoothing to a uniform 8mm Full Width Half-Maximum (FWHM) resolution, and only “raw” scans in their native resolution were used. Briefly, all PET images were co-registered to their matching T1W MRI. The T1W MRIs were spatially normalised to the MNI template with SPM8. The transform was then applied to the raw PET images. All the spatial normalisations were visually checked and failed registrations corrected using different parameters for the initial rigid/affine registration. A brain mask is applied to all spatially normalised images to remove the contribution of non-cortical voxels and reduce the memory footprint when training the model.

### Model

In the proposed model, DeepSUVR estimates a SUVR correction factor (CF) given a spatially and SUVR normalised image (where the image intensity is divided by the uptake in the whole cerebellum) and the corresponding tracer used. The network is based on our previous work (Yu et al., 2021) and is illustrated in supplementary Figure 17. The model includes four convolutional blocks, each with a 3D convolution (with increasing channel numbers from 16 to 128, kernel size=4 and stride=4), followed by instance normalisation and LeakyReLU. The output of the last convolutional block is flattened and concatenated with the tracer information, which is encoded as a one-hot vector of length 5. The first two fully connected blocks include a dropout with a rate of 0.5, a fully connected layer, batch normalisation and Tanh. The last layer includes a fully connected layer followed by a sigmoid to generate the CF. The sigmoid ensures that CF is within the [0,1] range. Lastly, to allow a full range of SUVR corrections, CF is multiplied by 2 to be constrained to the [0,2] positive range.

### Training strategy

The training set was split into 5 subsets for a 5-folds cross-validation. The subsets were balanced so that each fold had similar distribution of tracers, number of visits and Centiloid values and each participant could only belong to a single subset. The other 10 datasets were kept as independent test sets.

For each participant of the combined AIBL/ADNI dataset, pairs of PET images which were acquired at least 3 months apart, but no more than 3.5 years apart were selected – shorter timeframes tend to introduce too much noise, while longer timeframes can potentially bias the estimate of the Ab accumulation curve due to its non-linear nature. When three or more images were available within that timeframe, all permutations were considered (i.e. for three visits within 3.5 years, we would include the pairs [1–2], [2–3] and [1–3]).

**Figure 5:**
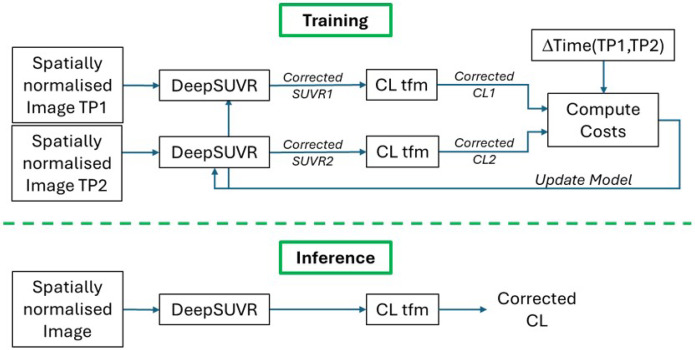
Framework used for training (top) and inference (bottom) using DeepSUVR. While training requires pairs of images from the same participant, inference is run on single images.

The training strategy for DeepSUVR is illustrated in Figure 5. For each pair, both images and their respective tracer information were run in succession through the DeepSUVR model during the same iteration, generating two *CFs*. The order in which the images were run through the network was randomised to ensure that it could not be learnt.

Data augmentation included random rotations (max 5 degrees) and non-rigid deformation (sigma=20, magnitude=50) to model small errors in spatial normalisation. It also included random smoothing to model differences in PET resolution from different scanners. The smoothness was constrained based on each scanner point-spread function (PSF), as measured using a Hoffman phantom, so that the image PSF after smoothing would not exceed 8mm FWHM. The data augmentation was implemented using the MONAI framework (Cardoso et al., 2022).

While longitudinal data are required to train the model, the model only requires a single image for inference. This means that DeepSUVR can be used to correct both longitudinal and cross-sectional studies.

### Loss function

For each pair, the loss function takes as input the standard SUVRs and their corresponding CF as estimated by the DeepSUVR model. It first computes the corrected SUVR as CF*SUVR and converts it into a corrected Centiloid using the standard and previously published Centiloid transforms (Bourgeat et al., 2018). Using those corrected Centiloid values, four loss functions are computed.

**Figure 6:**
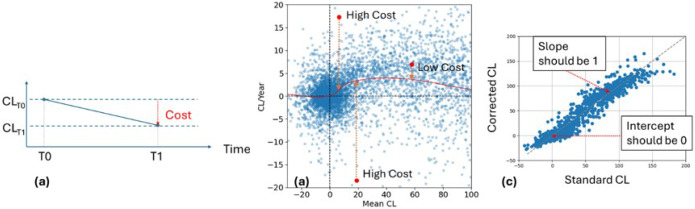
The penalties in the loss functions used to constrain the model: for each pairs of TPs, penalise the Centiloid decreasing over time (a); penalise the distance to the population curve of Mean CL vs Rate of change (b); for each batch, penalise the regression line of the standard CL vs corrected CL when it deviates from slope=1 and intercept=0.

- $L_{d}$-- Penalises the Centiloid decreasing over time (Figure 6.a): In an observational study, the Centiloid should always remain constant or increase over time but should not be decreasing. Therefore, we penalise decrease in Centiloids, with the loss being directly proportional to the amount of decrease. If the Centiloid remains constant or increases, then the loss is set to 0. Given two corrected Centiloid values $CL_{T0}$ and $CL_{T1}$ acquired at time T0 and T1 with T1>T0, the loss is defined as

$$○L_{d}=max\left(0,CL_{T0}-CL_{T1}\right)$$

- $L_{c}$-- Penalises deviations from the expected rate of change curve (Figure 6.b): As a pre-requisite, we need to first establish the reference curve that describes the relationship between the Centiloid value and its rate of change. This is performed by first computing for each pair of timepoints in the combined AIBL/ADNI dataset the mean Centiloid and the rate of change (in CL/Year) using the standard Centiloid quantification. To reduce variability due to the use of different tracers, this is restricted to PIB scans only. A locally weighted scatterplot smoothing (lowess) $f_{c}$ with a smoothing parameter=0.2, is fitted and used as a proxy to describe the expected rate of change for a given Centiloid value (Figure 6.b). Using the corrected Centiloid values, we compute the mean and rate of change. The absolute difference between the actual rate of change and the expected rate of change given the mean corrected Centiloid value is used to penalise unexpected changes in Centiloids over time, so that

$${○L}_{C}=/\left(CL_{T1}-CL_{T0}\right)/(T1-T0)-f_{C}\left(\left(CL_{T1}+CL_{T0}\right)/2\right)/$$

- $L_{s}$ and $L_{i}$-- Penalises the Centiloid deviating from the uncorrected Centiloid value (Figure 6.c). Without extra constraints, there is no penalty for the model over or under-correcting the Centiloids. In the extreme case, it could collapse all correction factors to 0. To ensure that the corrected Centiloids still represent meaningful quantities, we compute a regression line between the corrected and uncorrected Centiloid value for the entire batch, with the constraint that the slope should be 1 and the intercept should be 0, therefore ensuring that the Corrected Centiloids are still meaningfully associated with the uncorrected Centiloids. Given the slope s and the intercept i of the regression line between the corrected and uncorrected Centiloids, the losses are defined as

$$○L_{s}=\left|s-1\right|\;and\;L_{i}=|i|$$

- The four losses are combined in a single loss with weights a,b,g that were set empirically as a compromise between getting the best fit to the curve while minimising deviations from the standard Centiloids:

$$○L=L_{d}+aL_{c}+b\;L_{s}+g\;L_{i}$$

### Training

The model was trained on a H100 Nvidia card using a batch size of 128. The input images had a size of 91×109×91 with a 2mm isotropic voxel spacing. The weights for each loss were set to a=0.2, b=1, g=0.01. The model was trained with early stopping, monitoring the validation loss. Training was stopped when the validation loss did not improve for 20 consecutive epochs. To reduce the risk of the model converging to a suboptimal local minimum, for each fold, the model was trained using five different weight initialisations and dataloader shuffling, and the model with the highest Spearman rank correlation between the mean CL and rate of CL change in the validation set was selected as the best model.

### Generating new Centiloid masks

Deep learning models are often known for being black boxes, and their interpretability can be extremely challenging. In this work, we reformulate the explainability problem with the hypothesis that the corrections provided by DeepSUVR reflect changes in either the centiloid reference or target mask, or both. To validate this hypothesis, we here aim to optimise new reference and target masks that maximise the correlation with the corrected SUVR values. This is formulated as an optimisation problem with a new reference and target mask being optimised across all tracers using the AIBL+ADNI training dataset so that the resulting SUVR maximise the Pearson correlation coefficient with the DeepSUVR-corrected SUVRs. This optimisation is solved using a gradient descent approach and the use of two losses:

- $L_{p}$ – Penalises low Pearson correlation coefficient. Using the updated masks, new SUVRs can be computed across the entire dataset. The correlation between these SUVRs and the Corrected SUVR obtained by DeepSUVR is used to compute the Pearson coefficient ${\text{R}}^{2}$. The resulting loss is defined as:

$${○L}_{p}=1-R^{2}$$

- $L_{b}$ – Promote binary masks. The two masks being optimised need to have continuous values to ensure that they can be derived throughout the optimisation process. To force the mask M towards binarity, values not being either 1 or 0 are penalised. Additionally, the loss is normalised by the number of non-zeros-voxels B in the brain mask as follow:

$${○L}_{b}=S(0.5-/\text{M}-0.5|)/\text{B}$$

- The Pearson loss $L_{p}$ is computed for each tracer separately, and the average across the five tracers is used in the total loss. The binarity loss $L_{b}$ is computed for the reference and target masks separately and their sum is added to the total loss. A weights δ was assigned to the binarity loss with its value determined empirically to ensure convergence. The total loss L is defined as:

$$○L=\left(L_{p(PIB)}+L_{p(NAV)}+L_{p(FBB)}+L_{p(FBP)}+L_{p(FMM)}\right)/5+\delta \left(L_{b(\text{Reference})}+L_{b(\text{Target})}\right)$$

The masks are initialised using the original Centiloid reference and target mask and smoothed using a 4mm FWHM Gaussian kernel. At each iteration, the masks are updated using the gradient information and smoothed again using the same 4mm FWHM Gaussian kernel to ensure spatial consistency. The masks are also mirrored to generate symmetric masks. The optimisation leverages pytorch’s autograd engine for automatic computation of the gradients. Losses weights were initially set to δ=5e-4 and increased by a factor of 100 after 20 epochs, once the model started to converge. Using an initial smaller weight for the binary loss is necessary, as increasing its weight too early prevents the model from leaving its initial state. The random smoothing augmentation used to train DeepSUVR was also employed to reduce the variability due to different scanner’s PSF. To facilitate convergence, the optimisation is run using a 3-level multiresolution approach, where the masks are first optimised using x4 downsampled images. The masks are then upsampled and the optimisation resumed with x2 downsampled images, before finally being optimised on the full resolution images. For each resolution level, the optimisation is stopped once the improvement in $L_{p}$ is less than 10e-8 and the model has trained for a minimum of 1000 iterations at level 1, 3000 at level 2 and 8000 at level 3 (those were defined based on training without data augmentation, where the loss function is smooth and allowed to fully converge). Once the final masks are obtained, the new SUVRs derived from these masks are recalibrated into Corrected SUVR using a linear regression against the DeepSUVR-corrected SUVRs for each tracer, before being transformed into Centiloids using the standard transforms.

#### Evaluation

##### Cross-sectional analysis

We first evaluated the correlation between the Centiloids values derived from DeepSUVR $\left({\text{CL}}_{\text{DS}}\right)$ and those from the Standard method ${(\text{CL}}_{\text{std}})$ for each tracer and in both the training and testing datasets using the coefficient of determination $\left({\text{R}}^{2}\right)$.

The correlation between PIB Centiloid values and those from each of the ^18^F tracers within the GAAIN calibration dataset was assessed using ${\text{R}}^{2}$. A similar correlation analysis was performed for PIB and FBP in the OASIS dataset, specifically using matched scan pairs.

To assess the variability of the Aβ-negative across tracers and studies, we fitted a 2-Gaussian mixture model to each Centiloid distribution. The average of the mean and standard deviation of the first peak across all studies/tracers was then calculated.

To evaluate the stability of each method in estimating the mean and standard deviation of the first peak across studies and tracers, we performed a paired bootstrap analysis (N=10,000). For each bootstrap iteration, we computed the standard deviation of the peak means and peak standard deviations across studies or tracers for each method. We then calculated the paired difference between each method and the reference method $\left(\text{C}{\text{L}}_{\text{Std}}\right)$ for each bootstrap. The distribution of these paired differences was used to estimate the average difference in stability and a non-parametric p-value (based on the proportion of bootstrap samples with a sign opposite to the mean difference). A method was considered significantly more stable than ${\text{CL}}_{\text{Std}}$ if the 95% confidence interval did not include zero, corresponding to p<0.05.

The correlation between baseline Centiloid values and MMSE was evaluated using the Spearman rank correlation. We measured the effect size (Cohen’s d) for differences in Centiloid values between CDR scale categories: 0 vs 0.5, 0.5 vs 1 and 0 vs 1. The agreement between Centiloid values and visual reads in a subset of ADNI, ADNI-DOD, A4, HABS-HD and AMYPAD were quantified using the area under the receiver operating characteristic curve (AUC). Furthermore, we used Cohen’s d to measure the effect size of Centiloid differences between CERAD neuritic plaque score categories of “none/sparse” and “moderate/frequent”.

To assess whether any method provided a significant improvement over ${\text{CL}}_{\text{Std}}$ for each of the above-mentioned comparisons (correlation with MMSE, effect size between CDR levels, AUC with visual reads, and effect size with CERAD categories), a paired bootstrap (N=10,000) was conducted to generate non-parametric p-values.

##### Longitudinal trajectories

For studies with longitudinal data, we identified the number of longitudinal outliers based on the distribution of annual amyloid accumulation rates. The threshold was set at the 90^th^ percentile of the rate of change observed in AIBL study participants who underwent PiB PET scans exclusively on the same scanner (N=168; AD=20, CU=92; MCI=55). This approach established a reference range for Centiloid/year of [−5.8, 11.2]. Outliers were defined as consecutive visits of the same participant showing a decrease larger than 5.8CL/Year or an increase larger than 11.2CL/Year. The Hilbert-Schmidt Independence Criterion (HSIC) and Spearman ρ were used to assess the correlation between the mean Centiloid and the rate of change between consecutive pairs of visits acquired at least 3 months apart. While Spearman ρ is non-optimal to capture non-monotonic relationships, it is more commonly used for assessing non-linear associations and is easier to interpret. Therefore, both metrics are reported.

Within the A4 study, the effect size of the difference in amyloid accumulation rates between the treatment and placebo arms between the 2-month and the 56-month visits was measured using Cohen’s d.

To assess whether any method provided a significant improvement over ${\text{CL}}_{\text{Std}}$ for each of the above-mentioned comparisons (HSIC and Spearman ρ between mean Centiloid and rate of change, Cohen’s d between rate of CL accumulation in A4 treatment and placebo arms), a paired bootstrap (N=10,000) was conducted to generate non-parametric p-values.

##### Assessing the risk of overfitting the data to the curve

To evaluate our approach, we first compared the DeepSUVR results to the Standard Centiloid in the test set of the AIBL-ADNI cross-validation dataset. Those results informed the construction of new target and reference masks. CL computed using the new DeepSUVR masks $\left({\text{CL}}_{\text{DS}\;\text{mask}}\right)$ were then calculated in the ten independent validation studies. These ${\text{CL}}_{\text{DS}\;\text{Mask}}$ values were then compared to the Standard Centiloid values that used the WCb as the reference region $\left({\text{CL}}_{\text{Std}}\right)$. Additional comparisons, detailed in the supplementary materials, were conducted against Standard Centiloid values using the Composite WM+WCb reference region for FBP $\left(\text{C}{\text{L}}_{\text{Comp}}\right)$, and against our previously developed data-driven approach based on the Non-Negative Matrix Factorisation $\left({\text{CL}}_{\text{NMF}}\right)$.

A critical component of our model is the loss function $L_{c}$, which constrains pairs of Centiloid values to align with an expected rate of change curve. This constraint introduces a potential risk of over-correcting the Centiloid values thereby masking actual inter-participant variability in amyloid accumulation trajectories. Although most of the existing literature has converged towards a unique trajectory, it is plausible that natural variations could be hidden by the noise and variability in the existing Ab quantification methods. More advanced quantification techniques, such as DeepSUVR, might potentially allow their identification. To assess if DeepSUVR would over-correct the Centiloid quantification in this scenario, we simulated a different trajectory in a subset of the population and retrained the model on the simulated data. To this end, 10% of the participants in each fold of the cross-validation were assigned an artificially accelerated rate of amyloid accumulation by halving the time interval between their scans. The model was then retrained using this simulated dataset. The difference in the trajectory between the two populations was then assessed in the out-of-folds simulated AIBL+ADNI, using both the ${\text{CL}}_{\text{Std}}$, and the newly trained DeepSUVR model.

## Supplementary Files

This is a list of supplementary files associated with this preprint. Click to download.

- RevisitingCentiloidsusingAIsupplement.docx

## Figures and Tables

**Table 1.** AUC values for agreement between visual PET reads and the Centiloid values from different quantification method across specified cohorts. The highest AUC for each cohort is highlighted in bold.

	A4	ADNI	ADNIDOD	HABS-HD	AMYPAD	AMYPAD
${CL}_{\text{Std}}$	0.919	0.973	0.915	0.989	0.962	0.974
${CL}_{\text{Comp}}$	0.951***	0.965	0.927	-	-	-
${CL}_{\text{NMF}}$	0.948***	0.972	0.928	0.993***	0.975***	0.974
${CL}_{DS}$	**0.954** ***	**0.978**	**0.942** *	**0.994** ***	**0.982** ***	**0.979**
Tracers	FBP	FBP/FBB	FBP	FBB	FMM	FBB
N	1772	138	230	1094	1264	994
Visual Positivity (%)	35.0	48.6	30.9	7.8	24.0	19.3

The statistical significance over ${\text{CL}}_{\text{Std}}$ based on bootstrapping is indicated using:

* p<0.05,

** p<0.01,

*** p<0.001.

**Table 2.** Percentage of outliers based on the 95% confidence interval of yearly change in ${\text{CL}}_{\text{Std}}$ measured in PiB in AIBL, when no change of tracer or scanner occurred.

	Training cohort		Testing cohort	
	Neg outliers (%)	Pos outliers (%)	Neg outliers (%)	Pos outliers (%)
${CL}_{Std}$	6.45	6.74	2.48	1.76
${CL}_{\text{Comp}}$	5.84	4.95	1.80	1.26
${CL}_{\text{NMF}}$	4.70	3.69	1.17	1.22
${CL}_{DS}$	**1.58**	**1.92**	**0.48**	**0.50**
